# Interference with lactate metabolism by mmu-miR-320-3p via negatively regulating GLUT3 signaling in mouse Sertoli cells

**DOI:** 10.1038/s41419-018-0958-2

**Published:** 2018-09-20

**Authors:** Li-li Zhang, Jing Ma, Bo Yang, Jie Zhao, Bin-yuan Yan, Yuan-qiang Zhang, Wei Li

**Affiliations:** 1 Department of Obstetrics & Gynecology, Baoji Center Hospital, Baoji, 721008 Shaanxi Province P. R. China; 2 Department of Traditional Chinese Medicine, Xijing Hospital, Fourth Military Medical University, Xi’an, 710032 Shaanxi Province P. R. China; 3 Department of Urology, Xijing Hospital, Fourth Military Medical University, Xi’an, 710032 Shaanxi Province P. R. China; 4 Department of Human Anatomy, Histology and Embryology, Fourth Military Medical University, Xi’an, 710032 Shaanxi Province P. R. China; 5 Department of Epidemiology, School of Public Health, Fourth Military Medical University, Xi’an, 710032 Shaanxi Province P. R. China

## Abstract

Disruption of the nursery function in Sertoli cells (SCs) by reducing lactate production, a preferred energy substrate for developed germ cells (spermatocytes and spermatids), is tightly associated with spermatogenic failure such as SC-only syndrome (SCOS). However, whether this complicated pathogenesis is regulated by certain miRNAs at the post-transcriptional level remain fascinating but largely unknown. Here we show for the first time that mmu-miR-320-3p was exclusively expressed in murine SCs and this expression was significantly induced in busulphan-treated murine testis. The most efficient stimulatory germ cell types for the induction of apoptosis-elicited mmu-miR-320-3p expression were meiotic spermatocytes and haploid spermatids. Functionally, forced expression of the exogenous mmu-miR-320-3p in SCs compromises male fertility by causing oligozoospermia and defection of sperm mobility. Mechanistically, mmu-miR-320-3p negatively regulates lactate production of SCs by directly inhibiting glucose transporter 3 (GLUT3) expression. Thus, dysregulation of mmu-miR-320-3p/GLUT3 cascade and consequently of lactate deficiency may be a key molecular event contributing the germ cell loss by SC dysfunction. Future endeavor in the continuous investigation of this important circulating miRNA may shed novel insights into epigenetic regulation of SCs nursery function and the etiology of azoospermia, and offers novel therapeutic and prognostic targets for SCOS.

## Introduction

Known as “nurse cells”, the Sertoli cells (SCs) play a crucial role on the nutritional support of germ cells (GCs) during the each stage of spermatogenesis. Because differentiating GCs become more specialized and their biochemical machinery is insufficient to fulfill their metabolic demands, SCs therefore react in response to different metabolic stimuli to maintain the energetic homeostasis within this confined seminiferous tubules. Compelling evidence shows that multiple signaling cascades are essentially involved in this process. The AMP-activated kinase, sensitive to the global energetic status; the hypoxia-inducible factors, sensitive to oxygen concentration; and the peroxisome proliferator-activated receptors, sensitive to fatty acid availability, are all these striking examples^1^. Moreover, disruption of key signaling pathways (e.g. inhibition of mTOR pathway by rapamycin treatment) causes substantial but reversible impairment in male fertility, reemphasizing the indispensible involvement of these pathways during spermatogenesis and rendering the molecular explanation for reproductive side effects of certain immunosuppressants and anticancer agents^2,3^.

On the other hand, GCs exist in an environment created by SCs, so paracrine signaling between these intimately associated cells must regulate the proliferation and apoptosis in GCs^4^. In this context, it has been suggested that dysfunction of certain genetic factors and consequent impairment of SC function (such as GC nursery and junction formation) may play causative roles in spermatogenic failure^5^. Indeed, deletion of gap junction protein connexin43^6^, single-nucleotide polymorphisms in the *SEPTIN12* gene^7^, or attenuation of heterogeneous nuclear ribonucleoprotein L in SCs^8^ are all associated with the high risk of GC death. Nevertheless, the molecular mechanisms controlling SCs function remain largely unknown.

MicroRNAs (miRNAs), a cluster of single-stranded noncoding RNA molecules regulating post-transcriptional gene silencing via directly binding the to the 3′-untranslated region (3′UTR) of target mRNAs, play essential roles in the modulation of diverse biologic processes. Accordingly, altered miRNA expression and failures in their recognition of target genes are very likely to contribute to multiple pathologies, including spermatogenic arrest^9^. Actually, specific deletion of SC-derived Dicer, a central component of the RNA interference machinery, severely impairs SC competence, causes loss of mature GCs, and thereafter leads to male infertility, reflecting that miRNAs in SCs are essential for normal spermatogenesis^10^. However, the expression, functions, and targets of miRNAs in mammalian SCs remain largely unexplored^11^.

Recent high-throughput analysis has identified hsa-miR-320c as one of the most upregulated miRNAs in testicular biopsies of SC-only syndrome (SCOS) patients^12^. In this study, we found a novel regulatory relationship between mmu-miR-320-3p (the murine homolog of hsa-miR-320c) and glucose transporter 3 (GLUT3). We demonstrated that mmu-miR-320-3p is exclusively expressed in mouse SCs and this expression is significantly induced upon GC deletion. Furthermore, GLUT3 dysregulation induced by forced mmu-miR-320-3p expression impairs lactate production and compromises male infertility. Given that lactate is the main energy source for developed GCs and GLUT3 is the key carrier shunting glucose to lactate within SCs, our systematic analysis sheds novel insights into epigenetic regulation of SC nursery function.

## Results

### Predominant expression of mmu-miR-320-3p in murine SCs

**Fig. 1:**
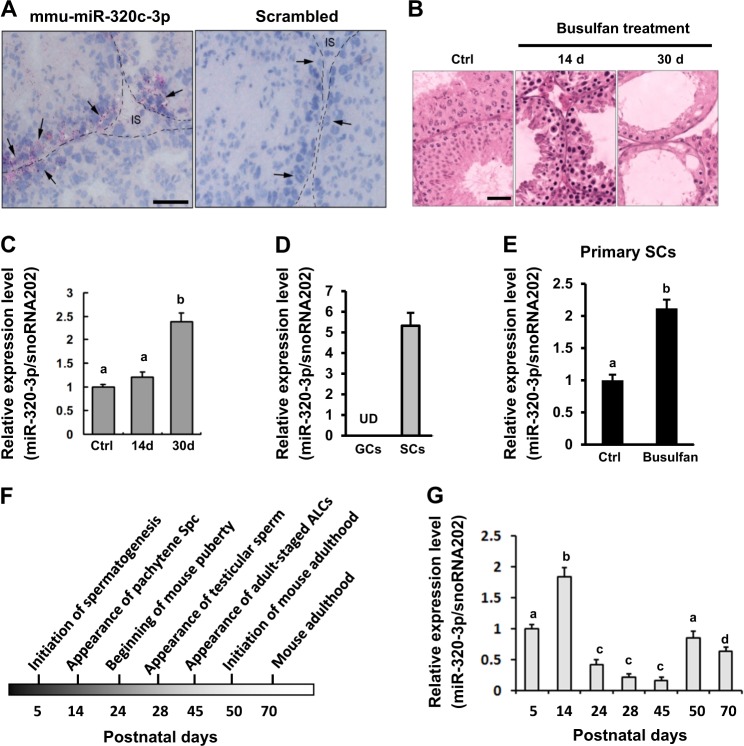
mmu-miR-320-3p is exclusively expressed in the Sertoli cells (SCs) of mouse testis. **a** Localization of mmu-miR-320-3p in the mouse testis was revealed by chromogenic in situ hybridization (CISH). Hybridization signals were in red (Fast-Red), and the nuclei were counterstained with Gill’s Hematoxylin (blue). Specific mmu-miR-320-3p hybridization signals (arrows) were exclusively detected in SCs, whereas the interstitium (IS) and germ cells (GCs) were both negative. Bar = 25 μm. **b** Effects of busulfan treatment on testicular histology were assayed using routine hematoxylin and eosin (H&E) staining. Bar = 20 μm. **c** Adult male mice were treated with busulfan as described in Materials and methods section. At post-busulfan d 14 and d 30, mice were euthanized, and testes were harvested and subjected to RT-qPCR analysis of mmu-miR-320-3p expression. Different superscript letters denote groups that are statistically different (*P* < 0.05). **d** RT-qPCR analysis of mmu-miR-320-3p expression in primary GCs and SCs isolated from normal mouse testis. Different superscript letters denote groups that are statistically different (*P* < 0.05). **e** RT-qPCR analysis of mmu-miR-320-3p expression in primary SCs isolated from busulfan-treated and Ctrl mouse testis. Different superscript letters denote groups that are statistically different (*P* < 0.05). (**f**) Diagram of the stages of murine spermatogenesis during postnatal development. **g** qPCR analyses of expression levels of mmu-miR-320-3p during postnatal testicular development. Different superscript letters denote groups that are statistically different (*P* < 0.05)

During the course of studying the potential post-transcriptional regulatory mechanisms in testicular tissues from infertile patients, we unexpectedly found that hsa-miR-320c-3p expression was significantly increased in testicular tissues from SCOS patients compared with that in the normal testes (Supplementary Fig. 1A and 1B, 1.57 ± 0.59 vs. 0.87 ± 0.36, *P* < 0.0001, Student’s *t*-test). To further study the functional involvement of miR-320c-3p in murine SCs, we determined the homology between mmu-miR-320-3p and hsa-miR-320c-3p. Sequence analysis revealed that mature mmu-miR-320-3p shared almost the same sequence of hsa-miR-320c-3p (including the seeding sequence, marked by dashed line box in Supplementary Fig. 1C). Next, we deciphered the expression pattern of mmu-miR-320-3p in mouse testis using chromogenic in situ hybridization (CISH). In situ hybridization with a mmu-miR-320-3p antisense probe revealed strong positive signals in SCs, whereas control experiments with a scrambled probe gave complete negative staining (black arrows in Fig. 1a). Not all the tubules were positively stained, indicating that mmu-miR-320-3p expression might be stage-specific. Because the testis comprises various cell types, we asked whether mmu-miR-320-3p exhibited cell type-specific expression in testis by examining the expression level of mmu-miR-320-3p in the testes from busulphan-treated mice. This cytotoxic drug induces apoptosis in spermatocytes within 2 weeks of treatment, and by day 30 most treated tubules contain only SCs (Fig. 1b, also see ref. ^5^). Interestingly, mmu-miR-320-3p expression was gradually stimulated along the busulphan treatment, with the highest value being detected at post-treatment day 30 [Fig. 1c, 2.34 ± 0.18 (day 30) vs. 1.20 ± 0.11 (day 14), *P* < 0.05; 2.34 ± 0.18 (day 30) vs. 1.20 ± 0.11 (Ctrl), *P* < 0.01], indicating that mmu-miR-320-3p is exclusively expressed in mouse SCs. To further confirm the above-observed phenotypes, we isolated GCs and SCs from busulfan-treated testis and Ctrl testis (Supplementary Fig. 2A), according to our previous work^5,13^. Compared to the abundant expression of mmu-miR-320-3p in SCs, mmu-miR-320-3p expression was undetectable in GCs, verifying the SC-specific expression of mmu-miR-320-3p (Fig. 1d). Moreover, in line with the in vivo data, busulfan treatment induced a significant increase of mmu-miR-320-3p expression by ~1.2-fold in primary SCs (Fig. 1e). Factors essential for SCs functions are usually expressed at distinct phases/stages along the testicular postnatal development^14^. Interestingly, quantitative PCR analyses using mouse testes corresponding to different developmental stages^15,16^ showed that mmu-miR-320-3p expression in mouse testis changed along the study period, with the highest values being detected during the pubertal period (day 24–45, Fig. 1f, g). Given that murine SCs are considered to be terminally differentiated during puberty^17^, our results indicate that miR-320-3p expression correlates well to the sufficient acquirement of SC function. Taken together, our data demonstrate that mmu-miR-320-3p is exclusively expressed in murine SCs and this expression appeared to be exhibited in a stage-specific and developmentally regulated pattern.

### Forced expression of exogenous mmu-miR-320-3p impairs male fertility in mouse

**Fig. 2:**
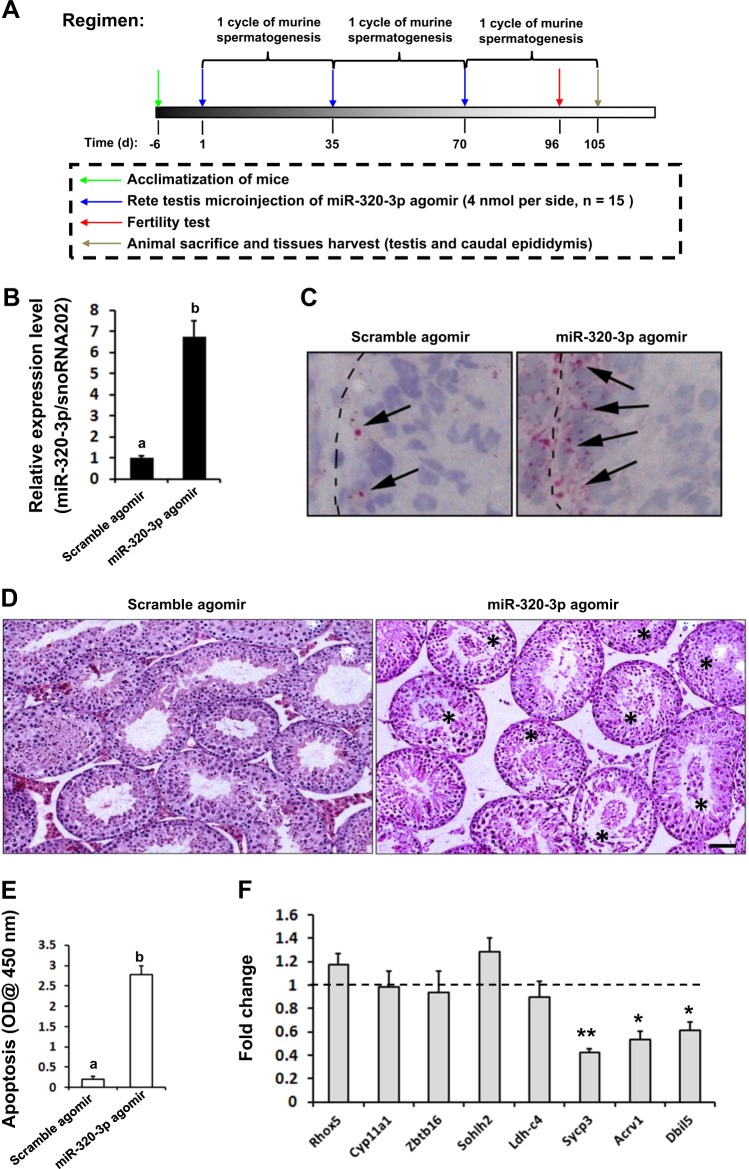
Ectopic expression of exogenous mmu-miR-320-3p impairs male infertility by causing apoptosis of distinct germ cells. **a** Schematic representation of the experimental procedure used in the in vivo agomir transfection study. After three cycles of mmu-miR-320-3p agomir injection as described in Materials and methods section, mice were euthanized and efficiency of mmu-miR-320-3p overexpression was verified using RT-qPCR (**b**) and CISH (**c**). **d** Effects of mmu-miR-320-3p overexpression on testicular morphology were determined using H&E staining. The asterisks denote seminiferous tubules that contained detached germ cells. Bar = 20 μm. **e** ELISA analysis of testicular apoptosis after the in vivo agomir transfection. Different superscript letters denote groups that are statistically different (*P* < 0.05). **f** The deleterious effects of mmu-miR-320-3p overexpression on the spermatogenic differentiation was evaluated by qRT-PCR analyses using specific primers for *Rhox5* (SCs), *Cyp11a1* (Leydig cells), *Zbtb16* (undifferentiated spermatogonia), *Sohlh2* (differentiating spermatogonia), *Ldh-c4* (preleptotene spermatocytes), *Sycp3* (pachytene spermatocytes), and for *Acrv1* and *Dbil5* (haploid spermatids), respectively. Results presented as mean ± S.E.M. of three independent experiments. *P < 0.05 and **P < 0.01 when compared to the values in Scramble agomir group

To directly ask whether mmu-miR-320-3p upregulation bears any functional consequence, we microinjected micrON™ mmu-miR-320-3p agomir or scrambled agomir into the rete testis (Fig. 2a) according to a previously validated protocol^18^. Mice receiving three cycles of microinjections exhibited a ~5.7-fold increase of testicular mmu-miR-320-3p expression compared to those injected with scrambled agomir (Fig. 2b). This forced mmu-miR-320-3p expression was further confirmed in situ by using CISH (Fig. 2c). Subsequent histological examination revealed that ~43.7% of the tubules contained desquamation of GCs upon mmu-miR-320-3p overexpression (Fig. 2d), suggesting a reduced spermatogenesis and a parallel testis degeneration. In keeping with these morphological defects, forced mmu-miR-320-3p expression caused an ~12.6-fold increase in testicular apoptosis (Fig. 2e, *P* < 0.05). To further identify at which stage the spermatogenesis was blocked, we performed qPCR analyses on testis RNA from mmu-miR-320-3p agomir- or scrambled agomir-treated mice using specific primers for *Rhox5* (SCs), *Cyp11a1* (Leydig cells), *Zbtb16* (undifferentiated spermatogonia), *Sohlh2* (differentiating spermatogonia), *Ldh-c4* (preleptotene spermatocytes), *Sycp3* (pachytene spermatocytes), and for *Acrv1* and *Dbil5* (haploid spermatids)^13,19^, respectively. All cell markers tested proved to be constantly expressed, with the significant decreases only being observable in the tetraploid spermatocytes and haploid spermatids of mmu-miR-320-3p agomir-treated mice (Fig. 2f). The available data made us wonder whether the surviving spermatozoa are capable of inseminating an egg, thereby retaining their reproductive competence. We carried out the mating experiments after completion of three cycles of murine spermatogenesis (Fig. 2a). The fertility potential was significantly compromised in mmu-miR-320-3p agomir-treated mice (reducing by ~75.0%), and oligozoospermia and reduction of sperm motility appeared to be account for this fertility impairment (Table 1). To assess the consequences of mmu-miR-320-3p overexpression on the barrier function of testis, we measured Evans blue dye uptake. Transfection with mmu-miR-320-3p agomir was unable to disrupt the testicular tight junctions, evidenced by an equal uptake of Evans blue dye between mmu-miR-320-3p agomir-treated testis and the testis treated with Scramble agomir (Supplementary Fig. 3a). Similarly, expression of TESTIN, a specific marker for the disruption of Sertoli-GC anchoring junctions^4^, was unaltered upon mmu-miR-320-3p agomir treatment (Supplementary Fig. 3B). These results together strongly suggested that a proper expression level of mmu-miR-320-3p in SCs is required for normal GC development.

**Table 1:** Assessment of male fertility and epididymal parameters after in vivo transfection assay

Experimental groups	Reproductive capacity			Characteristics of epididymal sperms	
	Pregnancies/females mated	Litter size	Number of males mated	Number of sperm (10^6^/epididymis)	Progressive motility (%)
Naive	41/46 (89.1%)	8.7 ± 2.4^a^	10	33.4 ± 1.7^a^	40.5 ± 3.3
Scramble agomir	38/41 (92.6%)	8.2 ± 1.3^a^	10	31.7 ± 0.9^a^	37.8 ± 1.8
miR-320-3p agomir	9/46 (19.6%)	3.4 ± 2.1^b^	10	13.6 ± 1.4^b^	12.9 ± 5.3^b^

Different superscript letters denote groups that are statistically different in the same category (*P* < 0.05)

### Supplement with sodium l-lactate rescues mmu-miR-320-3p-impaired male fertility

**Fig. 3:**
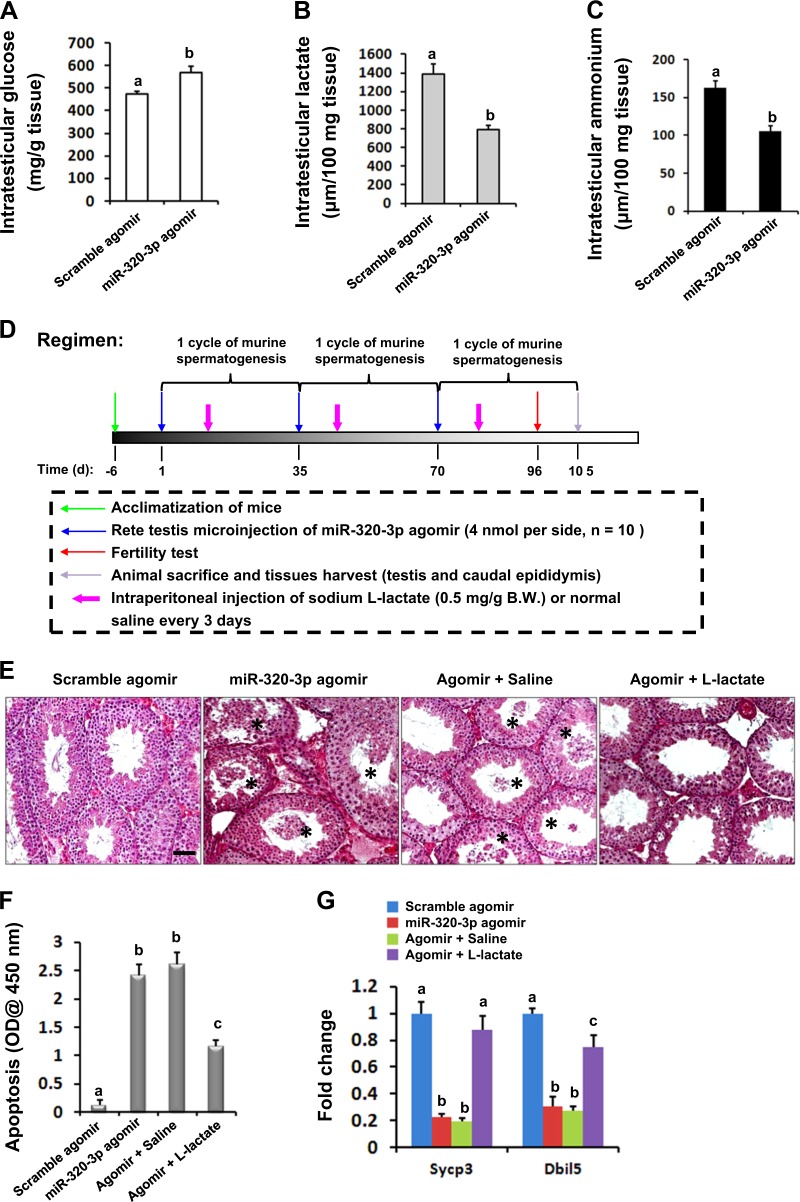
Sabotaging lactate production accounts for the impaired male infertility caused by mmu-miR-320-3p overexpression. After three cycles of mmu-miR-320-3p agomir injection as described in Materials and methods section, mice were euthanized and intratesticular levels of glucose (**a**), lactate (**b**), and ammonium (**c**) were determined using biochemical assays. Different superscript letters denote groups that are statistically different (*P* < 0.05). **d** Schematic representation of the experimental procedure used in the l-lactate supplementation study. **e** Effects of mmu-miR-320-3p overexpression, along with the supplementation with l-lactate, on testicular morphology were determined using H&E staining. The asterisks denote seminiferous tubules that contained detached germ cells. Bar = 20 μm. **f** Effects of mmu-miR-320-3p overexpression, along with the supplementation with l-lactate, on testicular apoptosis were evaluated using ELSIA. Different superscript letters denote groups that are statistically different (*P* < 0.05). **g** Effects of mmu-miR-320-3p overexpression, along with the supplementation with l-lactate, on testicular expression levels of *Sycp3* and *Dbil5* were assessed using RT-qPCR. Different superscript letters denote groups that are statistically different (*P* < 0.05)

Having established the causal link between mmu-miR-320-3p upregulation and testicular dysfunction, we then evaluated the intratesticular levels of glucose, lactate, and ammonium, given that the production of lactate and its metabolites by normal SCs is crucial for developing spermatocytes and spermatids^20^. Levels of intratesticular glucose were significantly stimulated (568.9 ± 29.64 vs. 473.7 ± 12.83, *P* < 0.01), whereas levels of intratesticular lactate (791.56 ± 47.64 vs. 1386.24 ± 106.88, *P* < 0.01) and of the metabolic waste product ammonium (a byproduct of glutaminolysis, 105.33 ± 7.61 vs. 163.27 ± 8.96, *P* < 0.05) were both notably reduced, in mmu-miR-320-3p agomir-treated mice (Fig. 3a–c). Thus, mmu-miR-320-3p upregulation in SCs may affect spermatogenesis by influencing lactate production. To validate this, we injected intraperitoneally sodium l-lactate or normal saline into mice along the treatment of mmu-miR-320-3p agomir or scrambled agomir (Fig. 3d). Histological examination after three cycles of co-treatment with mmu-miR-320-3p agomir and l-lactate supplementation revealed that replenishment of exogenous lactate could successfully prevent the mmu-miR-320-3p-induced detachment of GCs (Fig. 3e). Subsequent biochemical analyses further confirmed that this lactate replenishment successfully but partially rescued the development of spermatocytes and spermatids by reducing their apoptosis (Fig. 3f, g, 1.164 ± 0.105 for Agomir + l-lactate group vs. 2.607 ± 0.214 for Agomir + saline group, *P* < 0.05). In line with these laboratory examinations, the mating experiments confirmed from a functional angle that lactate injection could successfully rescue the fertility potential by ~1.5-fold in mmu-miR-320-3p agomir-treated mice (Table 2).

**Table 2:** Effects of in vivo mmu-miR-320-3p overexpression, along with the supplementation with l-lactate, on male fertility and epididymal parameters

Experimental groups		Reproductive capacity			Characteristics of epididymal sperms	
		Pregnancies/females mated	Litter size	Number of males mated	Number of sperm (10^6^/epididymis)	Progressive motility (%)
Scramble agomir		37/44 (84.1%)^a^	8.6 ± 1.5^a^	10	34.7 ± 2.5^a^	41.5 ± 5.4^a^
miR-320-3p agomir	Saline	15/51 (29.4%)^b^	3.2 ± 0.6^b^	10	16.9 ± 3.1^b^	19.1 ± 3.9^b^
	Sodium l-lactate	31/42 (73.8%)^a^	6.4 ± 2.3^a^	10	25.8 ± 1.9^c^	36.2 ± 6.1^a^

Different superscript letters denote groups that are statistically different in the same category (*P* < 0.05)

### Dysregulation of lactate metabolism by overexpression of mmu-miR-320-3p in TM4 SCs

**Fig. 4:**
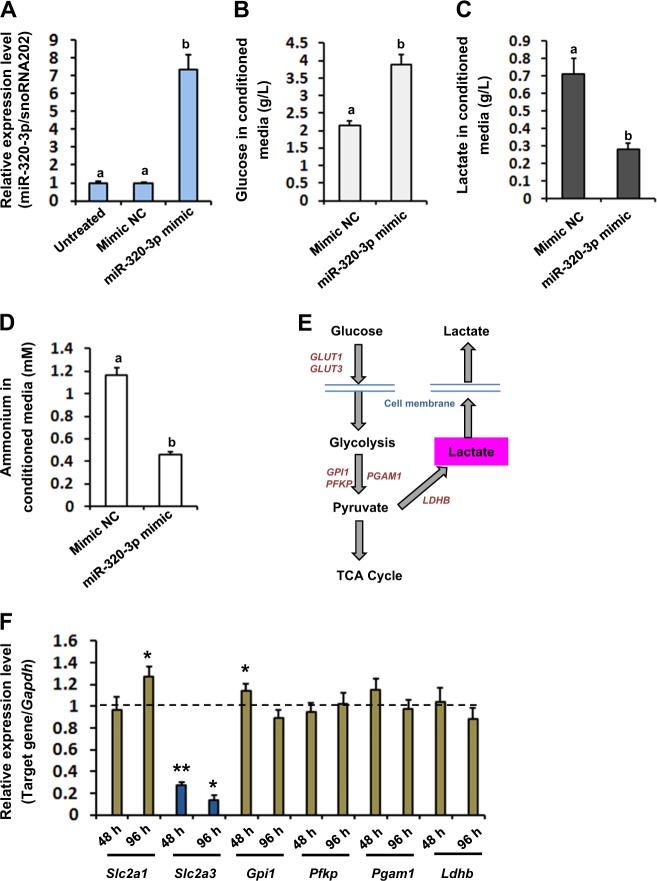
Forced mmu-miR-320-3p expression impairs lactate production in murine TM4 SCs. **a** Forty-eight hours after transfection with mmu-miR-320-3p mimic or negative controls (Mimic-NC), TM4 cells were collected and subjected to RT-qPCR analysis of the mmu-miR-320-3p expression. Different superscript letters denote groups that are statistically different (*P* < 0.05). Concentrations of glucose (**b**), lactate (**c**), and ammonium (**d**) in conditioned media from TM4 cells transfected with mmu-miR-320-3p mimic or Mimic-NC were quantified with Konelab Arena 60 automatic analyzer 48 h after transfection. **e** Schematic presentation depicting the main metabolic pathways important for lactate production. **f** Altered expression of genes impacting lactate metabolism in SCs transfected with mmu-miR-320-3p mimic or Mimic-NC were quantified by RT-qPCR. *P < 0.05 and **P < 0.01 when compared to the values in Mimic NC group

As further exploration of the functional meaning of mmu-miR-320-3p upregulation, we forced mmu-miR-320-3p expression by transfecting TM4 cells with mmu-miR-320-3p mimic or Mimic-NC (Fig. 4a). The production of lactate to fulfill the energy needs of developing GCs is a crucial function of SCs^20^. In our study, the concentration of lactate (0.283 ± 0.034 for mimic group vs. 0.712 ± 0.091 for Mimic NC group, *P* < 0.05), and the metabolic waste product ammonium during the synthesis of lactate (0.463 ± 0.021 for mimic group vs. 1.167 ± 0.066 for Mimic NC group, *P* < 0.01), was significantly lower in conditioned media from miR-320c-3p-overexpressed SCs as compared with controls, whereas the concentration of glucose (3.896 ± 0.275 for mimic group vs. 2.147 ± 0.134 for Mimic NC group, *P* < 0.05) was notably higher (Fig. 4b–d). We verified the deleterious effects of mmu-miR-320-3p overexpression on lactate metabolism in primary cultured SCs (Supplementary Fig. 2B-2E). Spurred by the metabolic results, we then investigated the expression of several marker genes known to sequentially appear along the conversion of glucose into lactate, at 48 h (short-term effect) and 96 h (long-term effect) after mimic transfection (Fig. 4e). Notably, enhancement of mmu-miR-320-3p expression was accompanied by increased *Slc2a1* expression (by ~27.4% for 96 h) and decreased *Slc2a3* expression at both time points (by ~72.8% for 48 h and ~86.4% for 96 h, Fig. 4f). Together, the available data suggest that mmu-miR-320-3p influences lactate production.

### Direct regulation of Slc2a3 expression by miR-320-3p

**Fig. 5:**
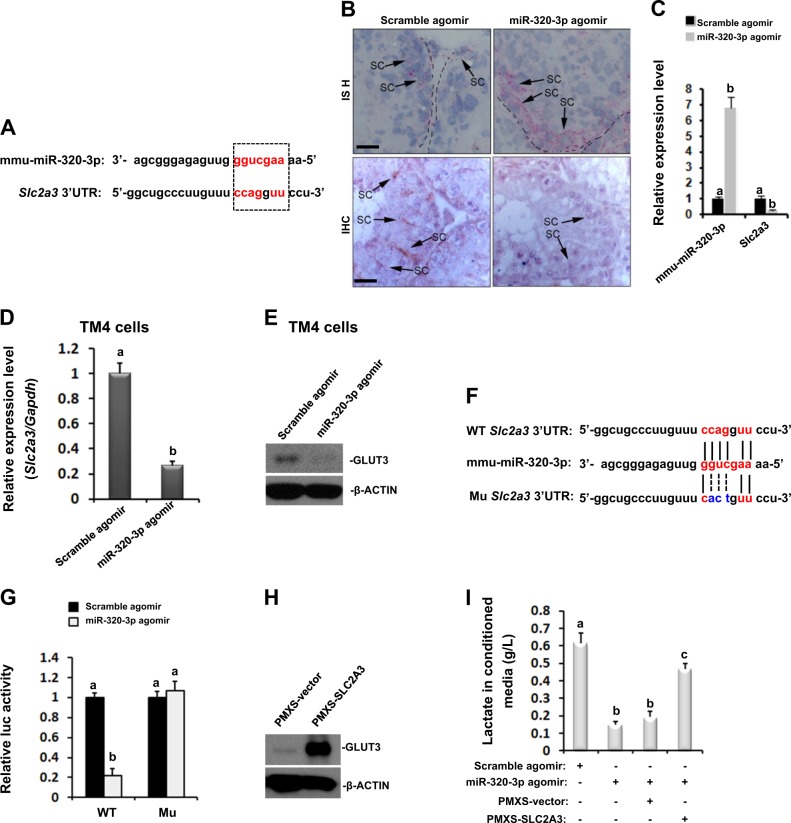
mmu-miR-320-3p directly targets GLUT3 by binding to the 3′UTR of Slc2a3 mRNA. **a** Schematic presentation showing the alignment of mmu-miR-320-3p mature sequence and the putative binding sites within the 3′UTR of *Slc2a3* mRNA using miRanda database. **b** Localizations of mmu-miR-320-3p and GLUT3 were visualized by CISH and immunohistochemistry in Scramble agomir- or miR-320-3p agomir-treated mouse testes, respectively. SC Sertoli cell, Spc spermatocytes, Spd spermatids. Bar = 10 μm. **c** RT-qPCR analysis of mmu-miR-320-3p and *Slc2a3* mRNA levels in Scramble agomir- or miR-320-3p agomir-treated mouse testes. Different superscript letters denote groups that are statistically different (*P* < 0.05). Forty-eight hours after transfection with mmu-miR-320-3p mimic or Mimic-NC, TM4 cells were subjected to RT-qPCR (**d**) and immunoblotting (**e**) analyses of CLUT3 expression. **f** An illustration of the construction of *Slc2a3*-3′UTR-WT and *Slc2a3*-3′UTR-Mu. **g** HeLa cells were co-transfected with 100 ng reporter plasmids, in the presence of miR-320-3p mimic or Mimic-NC using FuGENE^®^ 6. Twenty-four hours later, cells were subjected to luciferase activity measurements. **h** Verification of CLUT3 overexpression in TM4 cells by immunoblotting analysis. **i** Concentrations of lactate in conditioned media from TM4 cells with different transfections were quantified using Konelab Arena 60 automatic analyzer 48 h after co-transfection. Different superscript letters denote groups that are statistically different (*P* < 0.05)

Bioinformatics analysis revealed that the 3′UTR of *Slc2a3* (the gene encoding GLUT3) harbors one potential binding site for mmu-miR-320-3p (Fig. 5a). Regulation of *Slc2a3* expression by mmu-miR-320-3p was subsequently confirmed in three ways. First, mmu-miR-320-3p expression was demonstrated to be inversely correlated to the GLUT3 level in mouse testis using histochemistry and CISH (Fig. 5b), along with quantitative reverse transcription-PCR (RT-qPCR) analysis (Fig. 5c). Consistently, hsa-miR-320c expression was also inversely correlated to the *SLC2A3* mRNA level in clinical biopsies from SCOS patients (Supplementary Fig. 4A) and in semen samples from SCOS patients (Supplementary Fig. 4B). Furthermore, when TM4 cells were transfected with mmu-miR-320-3p mimic, GLUT3 expression was observed to be substantially inhibited at both transcriptional (Fig. 5d, 0.267 ± 0.034 for agomir group vs. 1.0 ± 0.083 for Scramble group, *P* < 0.05) and translational level (Fig. 5e). To investigate whether reduced *Slc2a3* expression was directly caused by mmu-miR-320-3p, we measured the luciferase activity after co-transfection of mmu-miR-320-3p mimic with *Slc2a3*-3′UTR-WT or *Slc2a3*-3′UTR-Mu in HeLa cells that are negative for GLUT3 expression (The Human Protein Atlas). The following reporter assay revealed that the luc activity was significantly suppressed by ~78.2% in HeLa cells co-transfected with *Slc2a3*-3′UTR-WT and mmu-miR-320-3p mimics, compared with Mimic-NC. In good contrast, mutation of the binding site by mmu-miR-320-3p in *Slc2a3*-3′UTR effectively abolished mimic-mediated suppression of luc activity (Fig. 5f, g). Lastly, we determined whether GLUT3 alone was sufficient to explain mmu-miR-320-3p-caused lactate deficiency. Transient transfection of TM4 cells with PMXS-SLC2A3 significantly increased GLUT3 expression (Fig. 5h), with no effects on mmu-miR-320-3p expression levels (Supplementary Fig. 5), reemphasizing that mmu-miR-320-3p acts upstream of GLUT3 signaling. More importantly, GLUT3 overexpression successfully but partially improved the lactate production in the presence of mmu-miR-320-3p mimic (Fig. 5i). Taken together, the results confirm the relevance of the disruption of the mmu-miR-320-3p/GLUT3 pathway in conferring lactate deficiency.

## Discussion

The data presented here indicate that miR-320-3p is induced in SCs upon GCs deletion, miR-320-3p induction compromises lactate production of SCs by directly inhibiting GLUT3 expression. Conversely, reintroduction of GLUT3 in SCs rescues lactate deficiency even in the presence of miR-320-3p overexpression. These data thus suggest that miR-320-3p mediates in part the nutritional support dysfunction in SCs caused by testicular metabolic disorders.

Alterations in cellular miRNA profiles have been previously reported in SCs. These miRNAs may be pivotal regulators of SCs growth, proliferation, and cytotoxicity^11^. Apart from intracellular miRNAs, recent advance in this field has also demonstrated the presence of miRNAs extracellularly in various body fluids. To this end, serum miRNAs have been found to be stable and readily detectable by RT-qPCR, which makes them attractive candidates for diagnosis, prognosis, and therapeutic targets under different pathological conditions^21^. miR-320c is such a striking example. Serum miR-320c has been found to be significantly upregulated during hepatitis C virus (HCV) infection and is proposed to play a regulatory role during the pathogenesis^21^. In the present study, we have identified mmu-miR-320-3p as a novel regulator of lactate metabolism in mouse SCs, and the expression level of hsa-miR-320c was significantly evoked in both testicular biopsies and semen samples from SCOS patients (Supplementary Fig. 4 and Fig. 5), suggesting that testing patients for hsa-miR-320c level may provide more accurate prognostic information for SCOS and may thereby influence the recommended course of treatment.

The mechanisms by which mmu-miR-320-3p expression is stimulated upon GC deletion remain to be revealed, but one factor has been so far reported to determine the level of mmu-miR-320-3p expression. As the only somatic cell type inside seminiferous tubules, SCs normally possess high metabolic rates, which make them often subjected to high oxidative stress levels that, if uncontrolled, may severely compromise SC nursery functions^22^. Compounds known to cause oxidative stress, such as Dibutyl phthalate^23^, aluminum^24,25^, and atrazine^26–28^, can impair the nutritional support of spermatogenesis by SCs. On the other hand, testicular oxidative stress has been shown to severely impair lactate dehydrogenase activity contributing low testicular lactate content^29^. However, how oxidative stress contributes to defective spermatogenesis via attenuation of SCs nursery function is still elusive. To this end, we may argue that the potential mechanism underlying oxidative stress action in SCs may, at least partially, be ascribed to control of oxidative stress-elicited activation of miR-320c-3p signaling. This intriguing possibility is currently being under investigation in our lab.

Whereas spermatogonia use glucose as a fuel for ATP production, more developed GCs, such as spermatocytes and spermatids, rely on SC-derived lactate as the main energy source. To ensure GC survival, SCs must ensure certain level of lactate production, even in adverse conditions^30^. Hence, it is important to recognize that absence of GCs may also be a reflection of underlying abnormalities in the SCs, such as failure of their lactate synthesis. Actually, insufficient supply of SC-derived lactate has been shown to be able to stimulate massive apoptosis of mature GCs^31^. In this regard, loss of GCs in SCOS may, at least partially, be caused by lack of enough lactate production in SCs. Our findings extend these understanding by identifying mmu-miR-320-3p as a potent upstream regulator of lactate metabolism in murine SCs. Two lines of observations may support this assumption: (i) both in vivo and in vitro assays confirmed that miR-320c-3p-overexpressed SCs produced decreased concentrations of lactate and the metabolic waste product ammonium (Figs. 3 and 4); and (ii) the glucose metabolism of miR-320c-3p-overexpressed TM4 SCs was attenuated as evidenced by a reduced expression of the key carrier (namely GLUT3) shunting glucose to lactate within SCs and decreased glucose utilization (Fig. 4f). Moreover, hsa-miR-320 has been shown to regulate glucose-induced gene expression in diabetes^32^. Proteomic analysis also indicates that hsa-miR-320a may regulate glycolysis broadly within nature^33^. Thus, modulation of glucose metabolism under different pathological conditions appears to be an intrinsic and conserved function in miR-320 family.

So far, expression of three glucose transporter isoforms, GLUT1, GLUT3, and GLUT8, has been observed in SCs. But only GLUT1 and GLUT3 are found to be expressed at the plasma membrane and are thereby believed to be the main executors transporting glucose from the extracellular milieu^30^. By using systematic analysis, we have observed that mmu-miR-320-3p could regulate GLUT3 expression by directly targeting its 3′UTR (Fig. 5). The interaction between mmu-miR-320-3p and *Slc2a3*-3′UTR was found to be specific, as mutating mmu-miR-320-3p seeding region in the 3′UTR of *Slc2a3* completely abrogated its regulatory effects (Fig. 5e, f). Our data corroborate that maintenance of GLUT3 activity is needed to promote lactate production in SCs. Interestingly, previous study has also identified GLUT3 as a bona fide target of hsa-miR-195-5p in human bladder cancer T24 cells^34^. Thus, GLUT3 appears to be a key regulator of glucose metabolism that seems to be targeted by different miRNA families, including the miR-320 family in our study. Of note, the compensative upregulation of GLUT1 expression could not reverse the deleterious effect of GLUT3 deficiency (Fig. 4f), reemphasizing the central regulatory role of GLUT3 in lactate metabolism of SCs.

**Fig. 6:**
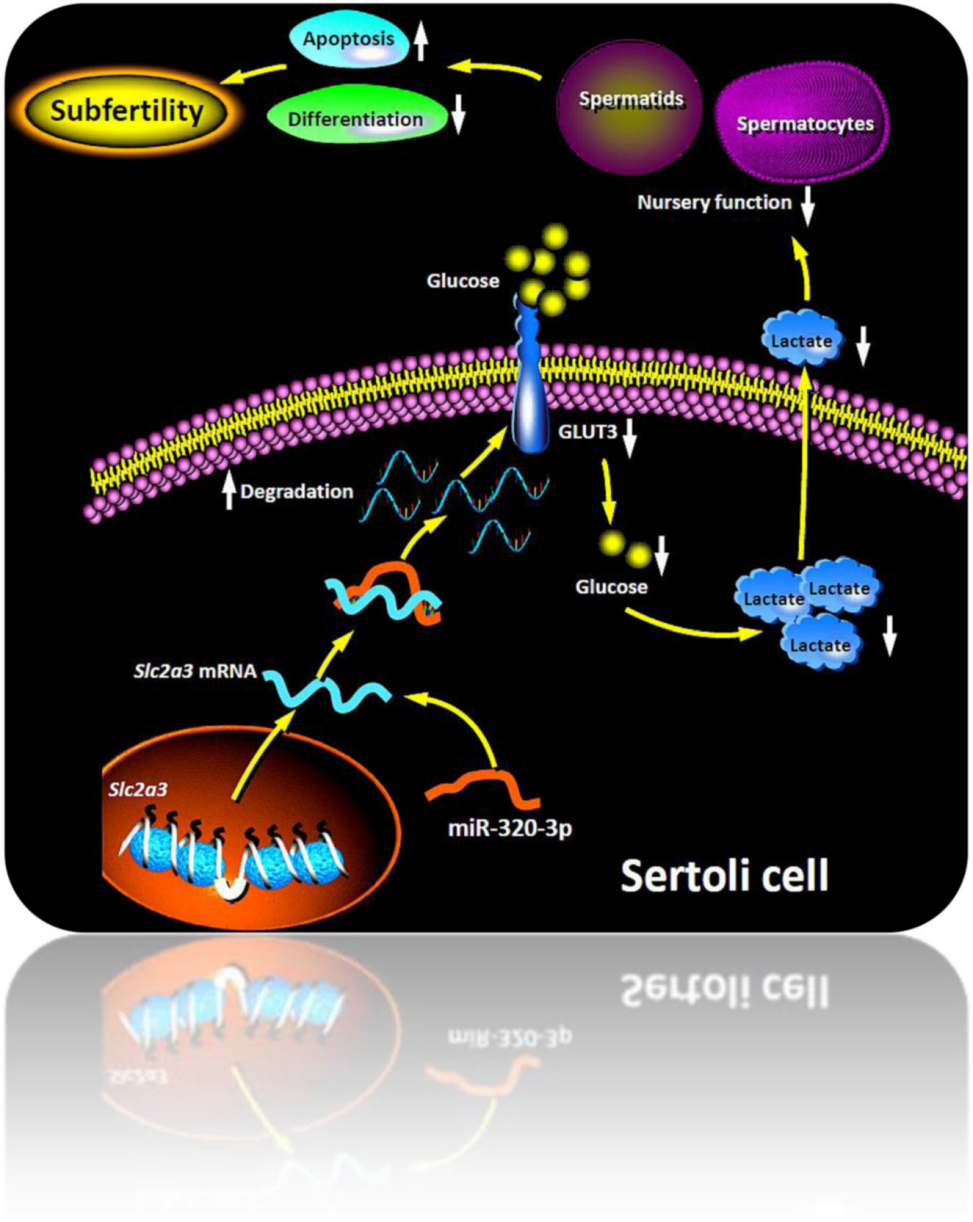
Summary diagram of the possible mechanisms related to testicular mmu-miR-320-3p function conferring lactate deficiency by directly regulating CLUT3 signaling pathway in SCs

In summary, the results obtained here show that SC-specific mmu-miR-320-3p expression is significantly stimulated in busulphan-treated murine testis. Forced expression of the exogenous mmu-miR-320-3p in SCs compromises male fertility by causing oligozoospermia and defection of sperm mobility. Mechanistically, mmu-miR-320-3p negatively regulates lactate production of SCs by directly inhibiting GLUT3 expression (Fig. 6). Thus, disruption of mmu-miR-320-3p/GLUT3 cascade and consequently of lactate deficiency may be a key molecular event contributing to the GC loss by SC dysfunction. The data in principle identify SCs miR-320-3p as a potential new therapeutic target for male infertility induced by nutritional support defects and spermatogenic metabolic dysregulation. Limitations of the current study lie in two aspects: (i) We need to further elucidate the potential regulation of miR-320-3p expression in SCs by oxidative stress signaling. (ii) A SC-specific knockout of miR-320-3p will be of great value to disclose the full involvement of miR-320-3p in the control of SC nutritional support function, in terms of the whole testis environment, and is therefore under progress in our lab.

## Materials and methods

### Animal model

Male BALB/c mice at different ages were obtained from the Animal Research Center of our university. Mice were maintained on a 12-h light:12-h dark in a 20–25 °C environment and they were allowed to acclimatize for at least 7 days before the experiment^35^. To eliminate the GCs, adult mice were given a single intraperitoneal (i.p.) injection of busulphan (30 mg/kg, Sigma-Aldrich, Shanghai, China) in dimethyl sulfoxide (DMSO, Sigma-Aldrich)/water (50/50 vol/vol) and were killed on postoperative day 14 or 30 (*n* = 5/group)^13^. Mice receiving only DMSO (50/50 vol/vol) were used as controls. All procedures in animal husbandry and handling, strictly sticked to the *Guide for Care and Use of Laboratory Animals* (NIH Publications No. 8023, revised 1978), were approved by the IACUC of Fourth Military Medical University.

### Histological work

For the routine morphological examination, testes were fixed in Bouin’s solution (Sigma-Aldrich) for 24 h and processed into 5-μm-thick sections for hematoxylin–eosin staining (Sangon Biotech, Shanghai, China).

Digoxigenin-labeled LNA-modified probe corresponding to mature mmu-miR-320-3p (sequence: 5′-AAAAGCUGGGUUGAGAGGGCGA-3′) and a scrambled probe (sequence: 5′-GTGTAACACGTCTATACGCCCA-3′) were prepared using miRCURY LNA™ miRNA ISH Optimization Kit (Exiqon, Woburn, MA, USA), as instructed by the manufacturer. Mice were perfused transcardially with 4% formaldehyde (4% PFA, Sangon Biotech), and then testes were removed, rapidly frozen in OCT Embedding Compound (Leica Biosystems, Beijing, China), cryosectioned for 10-μm thick, and subjected to CISH as described elsewhere^36^. Briefly, slides were treated with protease digestion (1:200 dilution, Thermo Fisher Scientific, Shanghai, China) for 30 min at 37 °C, followed by a thorough rinse using phosphate-buffered saline. Slides were then hybridized with 20 nM of miRNA in situ hybridization probe at 40 °C for 2 h, followed by incubation with Anti-Digoxigenin-AP antibody (Sigma-Aldrich) at room temperature for another 2 h. After a series of sequential washes using amplifier mix, the final chromogenic reaction was achieved using Fast-Red Substrate Kit (Abcam, Shanghai, China) for 30 min at 40 °C. Post-hybridized slides were postfixed with 4% PFA for 5 min at room temperature, counterstained with Gill’s Hematoxylin (Sigma-Aldrich), and mounted using Slide Mounting Media from Sigma-Aldrich.

To reveal the localization of GLUT3 in mouse testis, Avidin-Biotin complex (ABC) immunohistochemical method was conducted according to our previous work^18^, by employing the VECTASTAIN^®^ Elite^®^ ABC system (Vector Labs, Beijing, China). The primary antibody used was rabbit polyclonal anti-GLUT3 (1:200, Abcam).

### Reverse transcription-qPCR

Total RNA containing the small RNA fraction were isolated using MagMAX™ mirVana™ Kit (Thermo Fisher Scientific). Reverse transcription (RT) was performed by using miScript II RT Kit (QIAGEN, Shenzhen, China). For miRNA-specific qPCR, TaqMan® MicroRNA Assay kits (Applied Biosystems, Shanghai, China) were used and the reaction was carried out in an Applied Biosystems 7500 PCR System. Expression levels of other genes were determined by RT-qPCR using FAST-SYBR Green Master Mix (Thermo Fisher Scientific) according to the manufacturer’s specifications. Primers used for gene expression analysis are listed in Supplementary Table 1. Amplification of *Gapdh* or mouse snoRNA202 served as the internal control. Relative expression values from three independent experiments were calculated following the 2−△△Ct method^37^.

### In vivo overexpression of exogenous mmu-miR-320-3p

Adult BALB/c mice were anesthetized by i.p. injection of 2% pentobarbital sodium (50 mg/kg, Sigma-Aldrich). The testes were carefully pulled out from scrotum, and ~4 nmol of micrON™ mmu-miR-320-3p agomir or scrambled agomir (RiboBio, Guangzhou, China) was microinjected into the rete testis under an anatomical microscope (Leica Biosystems, Beijing, China). Mice were then returned for a 35-day break. In total, mice received three cycles of agomir treatment as indicated in Fig. 2a. To study whether inhibition of lactate alone can explain the mmu-miR-320-3p-induced disruption of male fertility, mice were intraperitoneally injected with sodium l-lactate (0.5 mg/g body weight) or normal saline every 3 days right after the first agomir injection. At 96th day after the first agomir injection, mice (*n* = 9) were subjected to fertility test as described below, and at 105th day, the rest of the animals were euthanized and testis and caudal epididymis were harvested for further evaluation of reproductive capacity.

### Assessment of male fertility and epididymal sperms

Fertility tests were carried out according to our previous work^5^. Briefly, males were individually housed with different wild-type (WT) females for 9 days, and were then set up again with other females for an additional 9 days, followed by record and calculation of pregnancy rate and litter size. Characterization of the morphology and motility of caudal epididymal sperms was performed as described in detail in our previous work^5^.

### Evaluation of testicular apoptosis

After animal sacrifice, testes were harvested and cytoplasmic fractions were isolated from tissues using ReadyPrep™ Protein Extraction Kit (Bio-Rad, Hercules, CA, USA). We then employed an apoptosis enzyme-linked immunosorbent assay kit to quantitatively measure cytoplasmic histone-associated DNA fragments (mononucleosomes and oligonucleosomes) (Roche Diagnostics, Shanghai, China). Final spectrophotometry was developed by a microplate reader at 405 nM (#680; Bio-Rad)^38^.

### Measurement of glucose, lactate, and ammonium concentrations in testis and conditioned culture media

Ten percent (w/v) homogenates from testis were prepared in pre-cold 6 N perchloric acid. The homogenates were then centrifuged at 1.0 × 10^4^ × *g* and the supernatant was then incubated with working glucose reagent at 37 °C for 10 min. The mixed solution was finally collected for evaluation of intratesticular glucose. In another experimental setting, 10% (w/v) testicular homogenates were prepared in pre-cold 0.5 M monophosphoric acid, followed by incubation with 5 M potassium carbonate solution at 4 °C for 20 min to neutralize the acid. The supernatants were finally collected for measurement of intratesticular lactate concentrations. Moreover, the testicular extracts were prepared for measurement of intratesticular ammonium concentrations using Ammonia Assay Kit, according to the manufacturer’s instructions (Abcam). The conditioned cell culture media that were collected 48 h after mimic transfection, along with the abovementioned tissue supernatants, were subjected to measurement of glucose, lactate, and ammonium concentrations using Konelab Arena 60 automatic analyzer (Thermo Fisher Scientific).

### Cell treatment

The murine SC line TM4 was obtained from American Type Culture Collection (Manassas, VA, USA) and cultured in Dulbecco’s modified Eagle’s medium (Invitrogen, Shanghai, China) supplemented with 10% fetal calf serum (Invitrogen). To overexpress the exogenous mmu-miR-320-3p, TM4 cells were transfected with mmu-miR-320-3p mimic or corresponding negative control (NC), using HiPerFect Transfection Reagent (Qiagen, Shanghai, China). Forty-eight hours after transfection, cells were harvested and subjected to other assays. To overexpress the exogenous GLUT3, TM4 cells were transfected with PMXS-SLC2A3, a gift from David Sabatini (Addgene plasmid # 72877, Cambridge, MA, USA)^39^, using HiPerFect Transfection Reagent (Qiagen). Forty-eight hours after transfection, cells were harvested and subjected to other assays.

### Immunoblotting

Immunoblotting was carried out according to our previous work^40^. Membranes were incubated with different primary antibodies, including rabbit anti-GLUT3 (Abcam) and rabbit anti-β-actin (Santa Cruz Biotechnology, Shanghai, China). β-actin served as loading control.

### Luciferase reporter assay

pLightSwitch-Luc-Blank and pLightSwitch-Luc-Slc2a3/3′UTR were purchased from SwitchGear Genomics (Shanghai, China). The site-directed mutagenesis of the miR-320a target site in Slc2a3/3′UTR was achieved using QuikChange Site-Directed Mutagenesis Kit (Agilent, Santa Clara, CA, USA), and designated as Slc2a3-3′UTR-Mu. For reporter assay, HeLa cells that are negative for GLUT3 expression (The Human Protein Atlas) were transfected with 100 ng reporter plasmids, in the presence of miR-320-3p mimic or Mimic-NC using FuGENE^®^ 6 (Promega, Madison, WI, USA). Twenty-four hours later, cells were subjected to luciferase activity measurements using LightSwitch Assay Reagents as instructed by the manufacturer.

### Statistical analysis

Results are presented as mean ± S.E.M. from at least three independent experiments. Data normality was determined using normal probability plots and compared using Student’s *t*-test. A *P*-value of <0.05 was considered statistically significant.

## Electronic supplementary material

Supplementary Figure legends

Supplementary Table 1

Supplementary Fig.1

Supplementary Fig.2

Supplementary Fig.3

Supplementary Fig.4

Supplementary Fig.5
