# Supplementary material for: Interference with lactate metabolism by mmu-miR-320-3p via negatively regulating GLUT3 signaling in mouse Sertoli cells
Source: Cell Death Dis. 2018 Sep 20;9(10):964. doi: 10.1038/s41419-018-0958-2 (PMC6148074; doi:10.1038/s41419-018-0958-2)
Supplement: Supplementary file 1 — Supplementary Figure legends [file 41419_2018_958_MOESM1_ESM.doc]

**Supplementary Figure legends**

**Supplementary Fig. 1** A total of 33 formalin-fixed paraffin-embedded testicular tissues from men with SCOS were included in this study. Testicular samples from men with obstructive azoospermia were used as Normaspermia group (n=20). Moreover, the semen samples from the above-mentioned SCOS patients (n=10) and from 8 normal healthy adult males were also collected for further biochemical analysis. All patients had a clinical diagnosis of azoospermia based on spermatozoa analyses and histopathological examination, and were referred to our Reproductive Medical Center for assisted reproduction between February and September 2012. The study was approved by the local Ethical Committee and all subjects gave written informed consent for participation according to the *Declaration of Helsinki*. (A) Histological examination of testicular tissues from SCOS patients and patients with normal spermatogenesis was carried out after H&E staining. (B) Relative expression level of hsa-miR-320c in SCOS or normal human testis was evaluated by RT-qPCR. (C) Schematic presentation showing the alignment difference between hsa-miR-320c mature sequence and mmu-miR-320-3p mature sequence. The dashed line box denotes the putative binding sites.

**Supplementary Fig. 2** (A) GCs and SCs were isolated and purified from mouse testis according to our previous work (see references 5 and 13). Values represent RT-qPCR analyses of RNA from purified mouse testicular cell populations with Sertoli, Leydig, and germ cell-specific markers, normalized to *Gapdh* using the comparative threshold cycle (^ΔΔ^CT) method. Different superscript letters denote groups that are statistically different (*P* < 0.05). UD, undetectable. (B) 48 h after transfection with mmu-miR-320-3p mimic or negative controls (Mimic-NC), primary SCs were collected and subjected to RT-qPCR analysis of the mmu-miR-320-3p expression. Different superscript letters denote groups that are statistically different (*P*<0.05). Concentrations of glucose (C), lactate (D) and ammonium (E) in conditioned media from primary SCs transfected with mmu-miR-320-3p mimic or Mimic-NC were quantified with Konelab Arena 60 automatic analyzer 48 hours after transfection. Different superscript letters denote groups that are statistically different (*P* < 0.05).

**Supplementary Fig. 3** Effects of *in vivo* miR-320-3p agomir treatment on the integrity of testicular tight junctions (TJs) or of anchoring junctions (AJs) were evaluated by measuring Evans blue dye uptake (A) and protein expression level of TESTIN (B), respectively.

**Supplementary Fig. 4** (A) *SLC2A3* mRNA level was inversely correlated to hsa-miR-320c expression in testicular biopsies from SCOS patients. (B) Comparison of hsa-miR-320c expression in semen samples from SCOS patients and normal controls by RT-qPCR.

**Supplementary Fig. 5** 48 h after transfection with PMXS-SLC2A3 or PMXS-vector, TM4 cells were subjected to RT-qPCR analyses of mmu-miR-320-3p expression level. Different superscript letters denote groups that are statistically different (*P* < 0.05)
