# Supplementary material for: Interference with lactate metabolism by mmu-miR-320-3p via negatively regulating GLUT3 signaling in mouse Sertoli cells
Source: Cell Death Dis. 2018 Sep 20;9(10):964. doi: 10.1038/s41419-018-0958-2 (PMC6148074; doi:10.1038/s41419-018-0958-2)
Supplement: Supplementary file 2 — Supplementary Table 1 [file 41419_2018_958_MOESM2_ESM.doc]

**Supplementary Table 1** Primers sets used for RT-qPCR analysis in the current study

| **Gene** | **Primer sequence** |
| --- | --- |
| *Rhox5* | F: CACCAGGACCAAAGTGGCC |
|  | R: GGTATGGAAGCTGAGGGTT |
| *Cyp11a1* | F: CCAGTGTCCCCATGCTCAAC |
|  | R: TGCATGGTCCTTCCAGGTCT |
| *Zbtb16* | F: CCTCACCAACCTTTCTT |
|  | R: ATCTTTGTCAGATCCATGA |
| *Sohlh2* | F: CAGCAAGACTCCTCAG |
|  | R: AGGAAGTACACAGACATC |
| *Ldh-c4* | F: CAGTTATAAACTCGCCAC |
|  | R: ATTGATCTTAACAGGTTTCC |
| *Sycp3* | F: AGCCAGTAACCAGAAAATTGAGC |
|  | R: CCACTGCTGCAACACATTCATA |
| *Acrv1* | F: TCAGCAACTTTCAAGCGAGTAT |
|  | R: CTCCTGAAGAGTGCTCACCTG |
| *Dbil5* | F: CCCAGGGCGACTGTAACATC |
|  | R: GCAATGTAGATCCTCATGGCAT |
| *Slc2a1* | F: TACACCCCAGAACCAATGGC |
|  | R: CCCGTAGCTCAGATCGTCAC |
| *Slc2a3* | F: GGGTGATGGTGGTTGATTGC |
|  | R: TCACCGAAAGCATCACAGCC |
| *Gpi1* | F: GGACGGCAAAGATGTGATGC |
|  | R: TGTCCGTGATGGATTTGCCA |
| *Pfkp* | F: CCCATGGTTATGGTTCCTGCT |
|  | R: GGTCGCACGTGTCTGTGATA |
| *Ldhb* | F: GGGTGAATGTGGCAGGAGTC |
|  | R: CAGCATGGACTCGATGAGGT |
| *Gapdh* | F: GGGTGAGGCCGGTGCTGAGT |
|  | R: TGACCCGTTTGGCTCCACCCT |
