# Supplementary material for: Interference with lactate metabolism by mmu-miR-320-3p via negatively regulating GLUT3 signaling in mouse Sertoli cells
Source: Cell Death Dis. 2018 Sep 20;9(10):964. doi: 10.1038/s41419-018-0958-2 (PMC6148074; doi:10.1038/s41419-018-0958-2)
Supplement: Supplementary file 3 — Supplementary Fig.1 [file 41419_2018_958_MOESM3_ESM.pptx]

## Slide 1
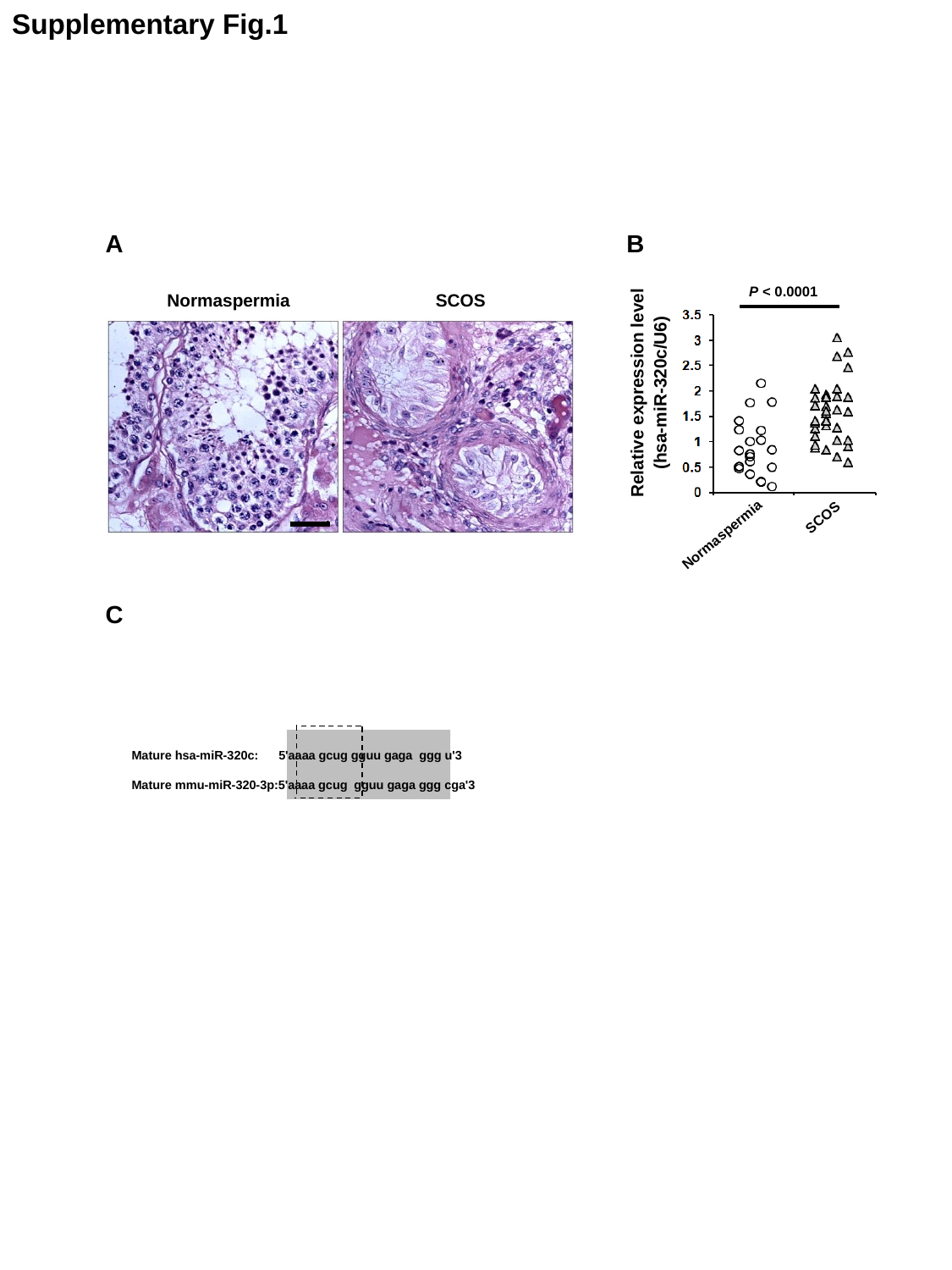

Supplementary Fig.1
A
B
P < 0.0001
Normaspermia SCOS
Relative expression level
(hsa-miR-320c/U6)
SCOS
Normaspermia
C
Mature hsa-miR-320c: 5'aaaa gcug gguu gaga ggg u'3
Mature mmu-miR-320-3p:5'aaaa gcug gguu gaga ggg cga'3
