# Supplementary material for: Interference with lactate metabolism by mmu-miR-320-3p via negatively regulating GLUT3 signaling in mouse Sertoli cells
Source: Cell Death Dis. 2018 Sep 20;9(10):964. doi: 10.1038/s41419-018-0958-2 (PMC6148074; doi:10.1038/s41419-018-0958-2)
Supplement: Supplementary file 4 — Supplementary Fig.2 [file 41419_2018_958_MOESM4_ESM.pptx]

## Slide 1
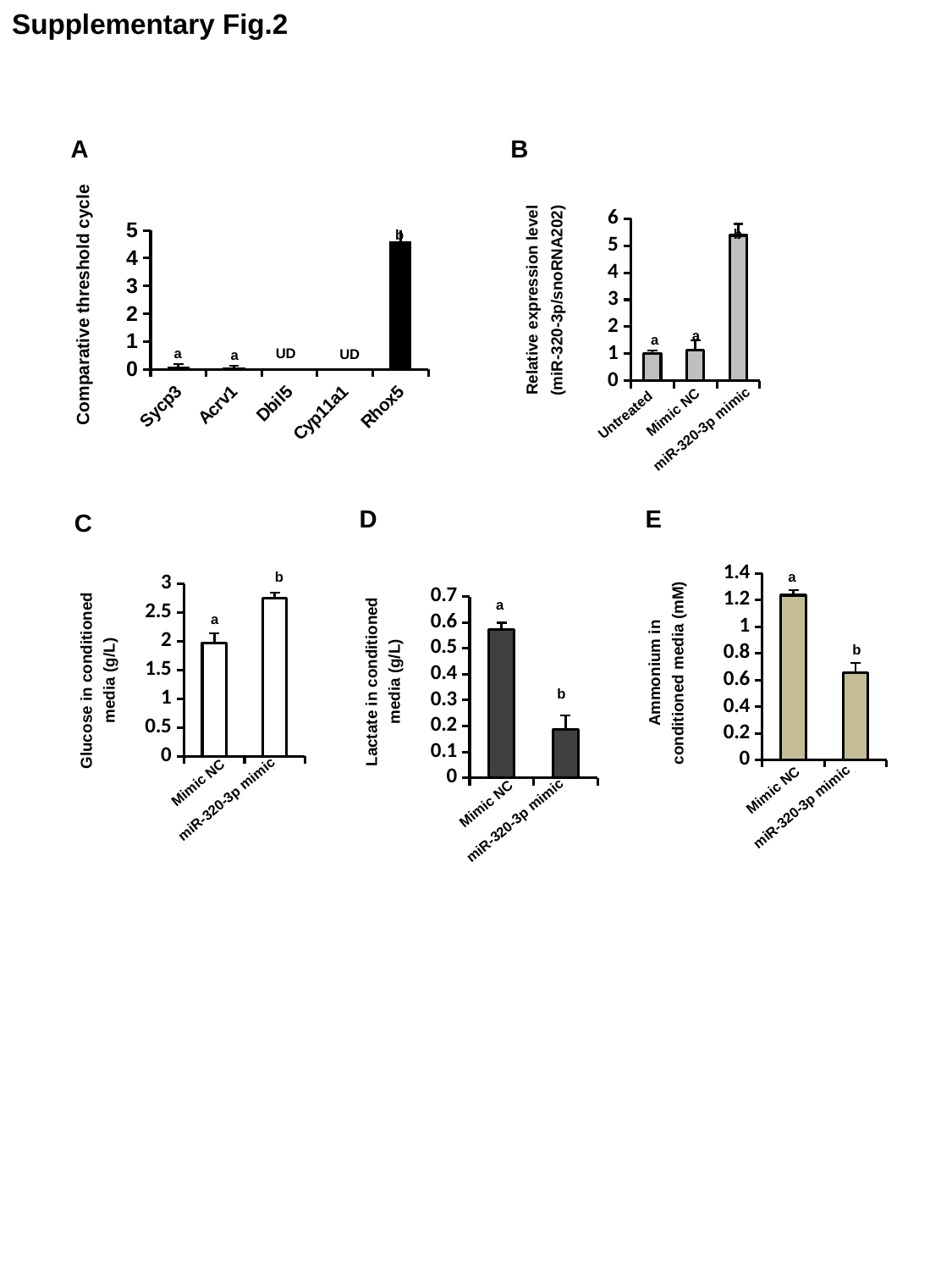

Supplementary Fig.2
### Chart
| Category | |
|---|---|A
B
### Chart
| Category | |
|---|---|
| Sycp3 | 0.10400000000000002 |
| Acrv1 | 0.082 |
| Dbil5 | 0.0 |
| Cyp11a1 | 0.0 |
| Rhox5 | 4.617999999999998 |b
b
Relative expression level
(miR-320-3p/snoRNA202)
Comparative threshold cycle
a
a
a
UD
UD
a
Untreated
Mimic NC
miR-320-3p mimic
### Chart
| Category | |
|---|---|
### Chart
| Category | |
|---|---|D
E
C
### Chart
| Category | |
|---|---|a
b
a
a
b
Ammonium in conditioned media (mM)
Glucose in conditioned media (g/L)
Lactate in conditioned media (g/L)
b
Mimic NC
miR-320-3p mimic
Mimic NC
miR-320-3p mimic
Mimic NC
miR-320-3p mimic
