# Supplementary material for: Interference with lactate metabolism by mmu-miR-320-3p via negatively regulating GLUT3 signaling in mouse Sertoli cells
Source: Cell Death Dis. 2018 Sep 20;9(10):964. doi: 10.1038/s41419-018-0958-2 (PMC6148074; doi:10.1038/s41419-018-0958-2)
Supplement: Supplementary file 5 — Supplementary Fig.3 [file 41419_2018_958_MOESM5_ESM.pptx]

## Slide 1
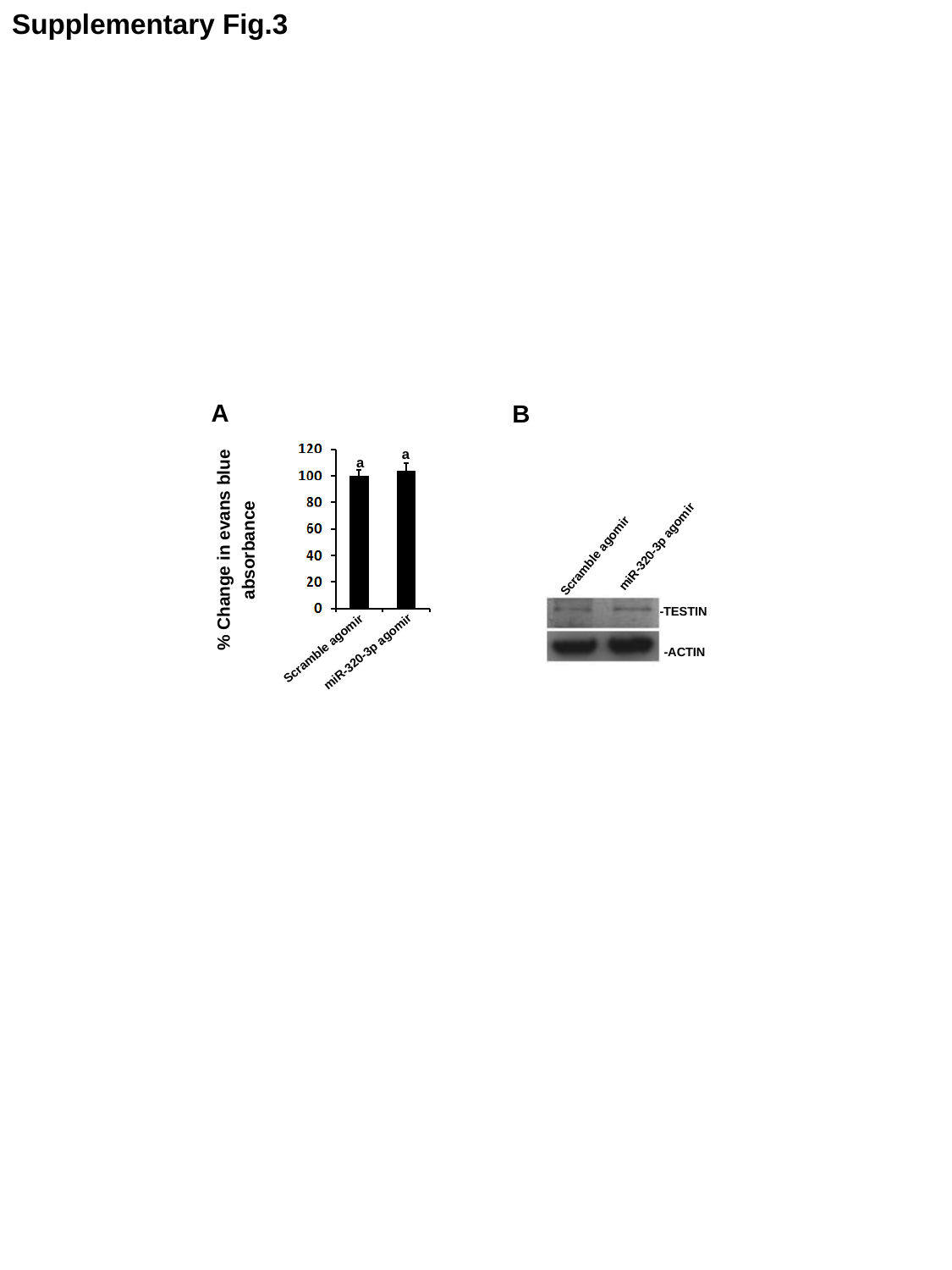

Supplementary Fig.3
A
B
a
a
% Change in evans blue absorbance
miR-320-3p agomir
Scramble agomir
-TESTIN
-ACTIN
Scramble agomir
miR-320-3p agomir
