# Supplementary material for: Interference with lactate metabolism by mmu-miR-320-3p via negatively regulating GLUT3 signaling in mouse Sertoli cells
Source: Cell Death Dis. 2018 Sep 20;9(10):964. doi: 10.1038/s41419-018-0958-2 (PMC6148074; doi:10.1038/s41419-018-0958-2)
Supplement: Supplementary file 6 — Supplementary Fig.4 [file 41419_2018_958_MOESM6_ESM.pptx]

## Slide 1
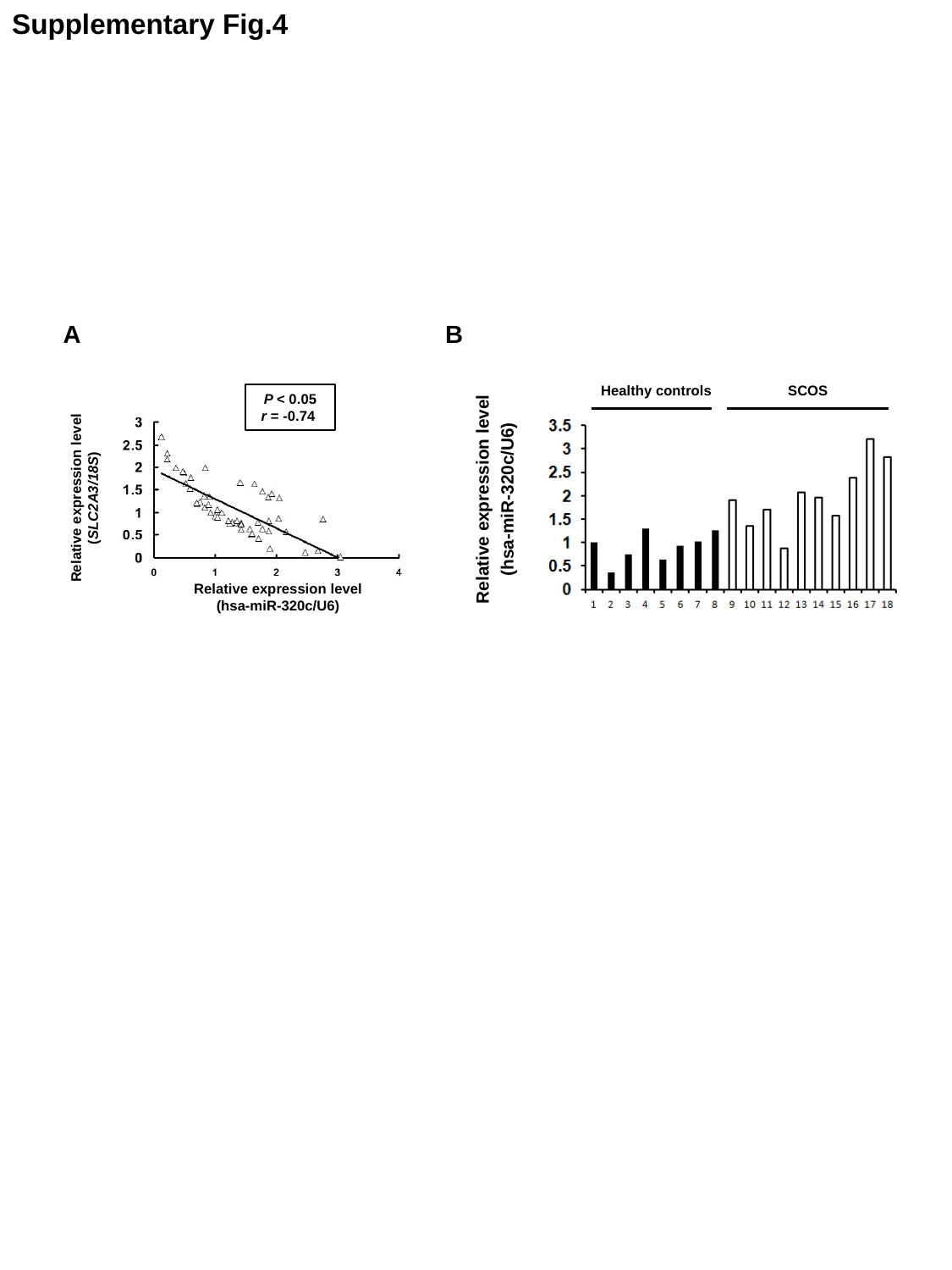

Supplementary Fig.4
A
B
Healthy controls SCOS
P < 0.05
r = -0.74
Relative expression level
(hsa-miR-320c/U6)
Relative expression level
(SLC2A3/18S)
Relative expression level
(hsa-miR-320c/U6)
