# Supplementary figures and images for: Interference with lactate metabolism by mmu-miR-320-3p via negatively regulating GLUT3 signaling in mouse Sertoli cells
Source: Cell Death Dis. 2018 Sep 20;9(10):964. doi: 10.1038/s41419-018-0958-2 (PMC6148074; doi:10.1038/s41419-018-0958-2)

## Slide 1
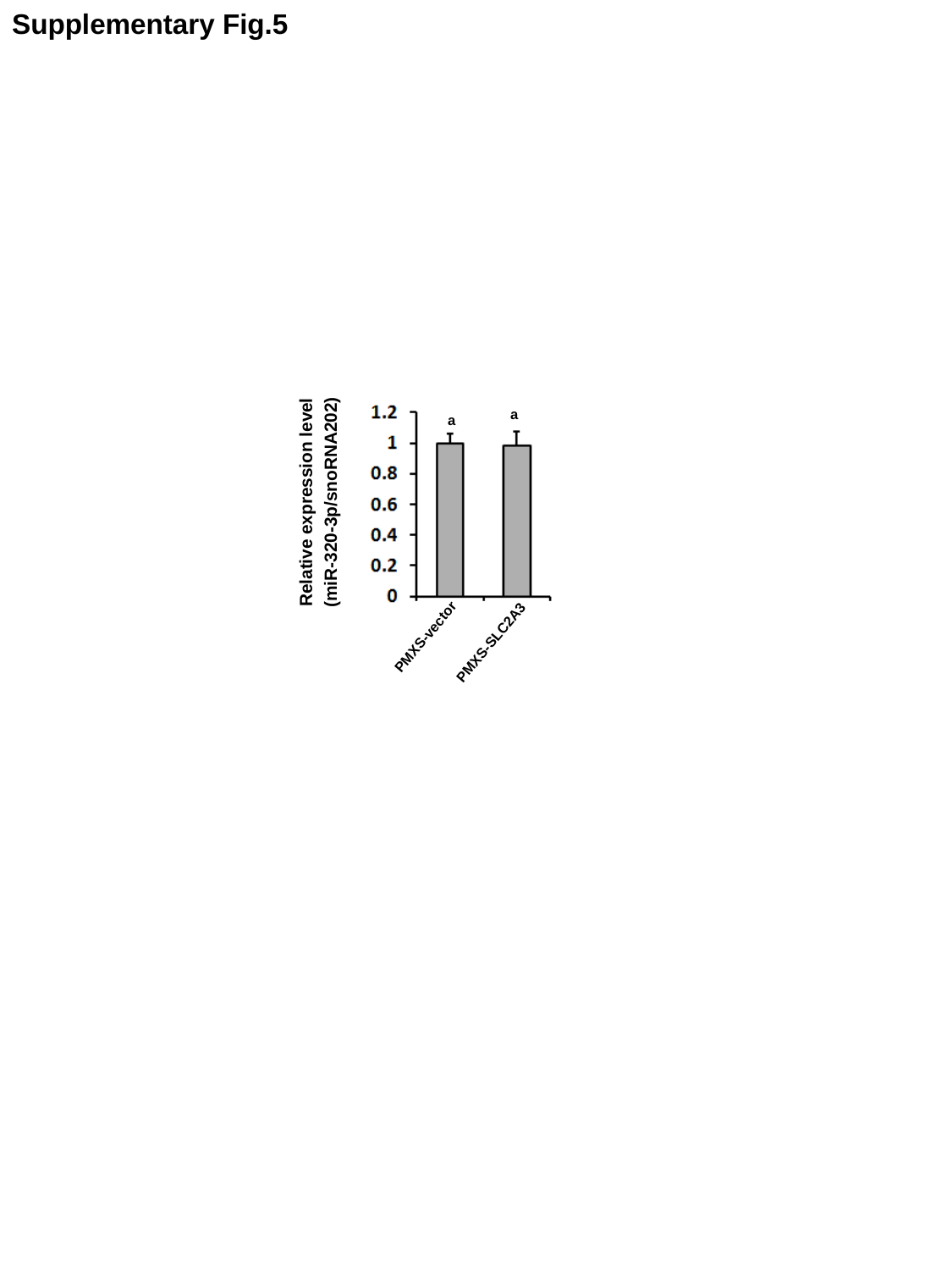

Supplementary Fig.5
a
a
Relative expression level
(miR-320-3p/snoRNA202)
PMXS-vector
PMXS-SLC2A3

Supplement: Supplementary file 7 — Supplementary Fig.5 [file 41419_2018_958_MOESM7_ESM.pptx]
